# Time-keeping and decision-making in the cell cycle

**DOI:** 10.1098/rsfs.2021.0075

**Published:** 2022-06-10

**Authors:** John J. Tyson, Béla Novák

**Affiliations:** ^1^ Department of Biological Sciences, Virginia Tech, Blacksburg, VA, USA; ^2^ Department of Biochemistry, University of Oxford, Oxford, UK

**Keywords:** cell cycle regulation, cell cycle checkpoints, cyclin-dependent kinases, mathematical models, limit cycles, bistable switches

## Abstract

Cell growth, DNA replication, mitosis and division are the fundamental processes by which life is passed on from one generation of eukaryotic cells to the next. The eukaryotic cell cycle is intrinsically a periodic process but not so much a ‘clock’ as a ‘copy machine’, making new daughter cells as warranted. Cells growing under ideal conditions divide with clock-like regularity; however, if they are challenged with DNA-damaging agents or mitotic spindle disrupters, they will not progress to the next stage of the cycle until the damage is repaired. These ‘decisions’ (to exit and re-enter the cell cycle) are essential to maintain the integrity of the genome from generation to generation. A crucial challenge for molecular cell biologists in the 1990s was to unravel the genetic and biochemical mechanisms of cell cycle control in eukaryotes. Central to this effort were biochemical studies of the clock-like regulation of ‘mitosis promoting factor’ during synchronous mitotic cycles of fertilized frog eggs and genetic studies of the switch-like regulation of ‘cyclin-dependent kinases' in yeast cells. In this review, we uncover some secrets of cell cycle regulation by mathematical modelling of increasingly more complex molecular regulatory networks of cell cycle ‘clocks’ and ‘switches’.

## 1. Introduction

**Figure 1.**
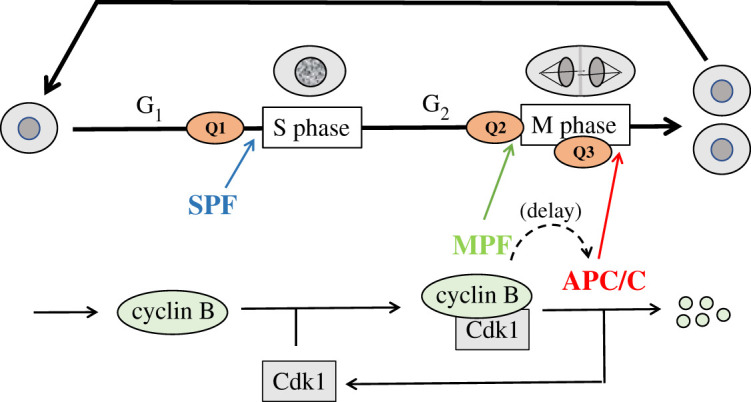
The cell cycle. Upper half: in general, the eukaryotic cell cycle is divided into four phases: G_1_ (unreplicated DNA), S (DNA synthesis), G_2_ (replicated DNA) and M (mitosis). During S phase, every chromosome is replicated, and during M phase, the ‘sister chromatids’ are pulled to opposite poles of the mitotic spindle, followed by cell division. Progression through the cell cycle is monitored at three checkpoints: Q1 (Is DNA undamaged?), Q2 (Is DNA fully replicated?) and Q3 (Are chromosomes properly aligned on the mitotic spindle?). If the answer to Q1 is yes, then S phase is initiated by its ‘promoting factor’ SPF. If the answer to Q2 is yes, then M phase is initiated by its ‘promoting factor’ MPF. If the answer to Q3 is yes, then anaphase is initiated by its ‘promoting complex’ APC/C. The dynamics of these promoting factors are the subject of this review. Lower half: in the first few hours after fertilization, a frog egg proceeds through rapid mitotic cycles. As cyclin B is synthesized, MPF activity (CycB : Cdk1) rises and initiates mitosis. At the end of mitosis, APC/C is activated, and cyclin B is rapidly degraded after a significant time delay. In the next cycle, when MPF activity is low, SPF drives DNA replication. These early embryonic cycles alternate between S phase and M phase, lacking gap phases and checkpoints.

The cell cycle is the sequence of events whereby a living cell replicates all its components and divides, more or less evenly, into two daughter cells that receive all the material and information necessary to repeat the process. The central role of the cell cycle is to transmit a cell's genome (its full complement of DNA molecules) to the next generation of cells. In eukaryotic cells, this role is achieved by fully and accurately replicating all chromosomes during S phase of the cell cycle, and subsequently by precisely partitioning the replicated chromosomes to the two daughter cells during M phase (mitosis) and cell division (figure 1, upper half). S and M phases are usually separated by temporal gaps, and the sequence G1–S–G2–M/CD is repeated faithfully in succeeding cell cycles [1].

The replication of cells is an intrinsically cyclic process because cell division creates two daughter cells that are primed to repeat the process. In some circumstances, like the early embryonic divisions of a frog egg or laboratory cells growing in a rich culture medium, cells replicate their DNA and divide periodically, like a clock; whereas in other circumstances, like cells whose DNA has been damaged by ionizing radiation, progression through the cell cycle is blocked by switch-like ‘surveillance and checkpoint’ mechanisms. Thus, control of cell cycle progression shares characteristics of the time-keeping and decision-making processes that are the theme of this special issue [2,3].

Because cell growth and division are foundational to all biological growth, repair, reproduction and development, it is crucial that we sort out the clock-like and switch-like properties of the cell cycle, and that we come to understand the molecular mechanisms that underlie these processes. Success in this endeavour will have far-reaching consequences for agriculture, medicine and the biotech industry.

## 2. Cyclin-dependent kinases

In eukaryotic cells, the initiation of cell cycle events and the detection and correction of errors are carried out by a complex network of interacting proteins centred around a class of enzymes called ‘cyclin-dependent kinases' (CDKs). By phosphorylating and activating (or inactivating) a host of target proteins, CDKs orchestrate the events of the cell cycle, and CDKs themselves are targets of ‘error correction mechanisms’ that block further progression through the division cycle until problems in the copying or partitioning of chromosomes can be resolved [1]. As their name implies, CDKs must bind to a regulatory subunit, a ‘cyclin’ molecule, in order to be able to phosphorylate specific protein targets. The first cyclin–CDK dimer to be characterized biochemically was cyclin B–Cdk1 [4], also known as M-phase promoting factor (MPF). We hereafter indicate dimers with a colon, e.g. CycB : Cdk1.

The biochemistry of MPF was unravelled in the 1970s and 80s by the joint efforts of biochemists, geneticists and molecular biologists. The basic picture that arose from early studies of MPF dynamics in frog oocytes and embryos [5] is illustrated in figure 1 (lower half). Cyclin B is synthesized at a constant rate and rapidly combines with a constant pool of Cdk1 subunits to form CycB : Cdk1 dimers (i.e. active MPF). When MPF activity is high enough, the cell enters mitosis. Among many other mitotic substrates, high activity of MPF phosphorylates and activates the anaphase-promoting complex/cyclosome (APC/C). The APC/C has two decisive functions [1]: to activate separase (promoting sister chromatid separation in anaphase) and to polyubiquitinate cyclin B (promoting degradation of cyclin B by proteasomes). APC/C activation is delayed to give the cell time to condense its chromosomes and align them on the mitotic spindle [6]. Then, APC/C activity is maintained long enough during anaphase and telophase to degrade most of the cell's cyclin B, even though MPF activity is dropping precipitously as cyclin B is degraded. These curious properties of APC/C activity—the time delay and the persistence—will play crucial roles in our story.

## 3. Cell cycle oscillations in early embryonic divisions of the frog egg

During divisions 2–12 of a fertilized frog egg, the embryonic cells divide rapidly and synchronously without growth or checkpoint mechanisms. Cyclin B accumulates steadily during interphase and is then rapidly and fully degraded as cells exit mitosis and divide. These oscillations are driven by periodic degradation of cyclin B, in consequence of the negative feedback loop by which CycB : Cdk1 activates APC/C [7], which in turn causes the degradation of cyclin B and loss of CycB : Cdk1 activity (figure 1, lower half).

### (a) Model no. 1

In 1991, Goldbeter [8] modelled these oscillations with a set of ordinary differential equations (ODEs) for [MPF], [Kin] and [APC]:

3.1a $$\begin{array}{r} \frac{d[\mathrm{MPF}]\;}{dt}=k_{\mathrm{sy},\mathrm{cycb}}-(k_{\mathrm{de}1,\mathrm{cycb}}+k_{\mathrm{de}2,\mathrm{cycb}}[{\mathrm{APC}}_{P}])\cdot [\mathrm{MPF}], \end{array}$$

3.1b $$\frac{d[{\mathrm{Kin}}_{P}]}{dt}=\frac{k_{\mathrm{ph},\mathrm{gwl}}[\mathrm{MPF}]\;[\mathrm{Kin}]\;}{J_{\mathrm{ph},\mathrm{kin}}+[\mathrm{Kin}]\;}-\frac{k_{\mathrm{dp}1,\mathrm{gwl}}[\mathrm{PP}]\;[{\mathrm{Kin}}_{P}]}{J_{\mathrm{dp},\mathrm{kin}}+[{\mathrm{Kin}}_{P}]}$$

3.1c $$\begin{array}{r} \mathrm{and}\;\frac{d[{\mathrm{APC}}_{P}]}{dt}=\frac{k_{\mathrm{ph},\mathrm{apc}}[{\mathrm{Kin}}_{P}]\;[\mathrm{APC}]\;}{J_{\mathrm{ph},\mathrm{apc}}+[\mathrm{APC}]\;}-\frac{k_{\mathrm{dp},\mathrm{apc}}[\mathrm{PP}]\;[{\mathrm{APC}}_{P}]}{J_{\mathrm{dp},\mathrm{apc}}+[{\mathrm{APC}}_{P}]}. \end{array}$$

**Figure 2.**
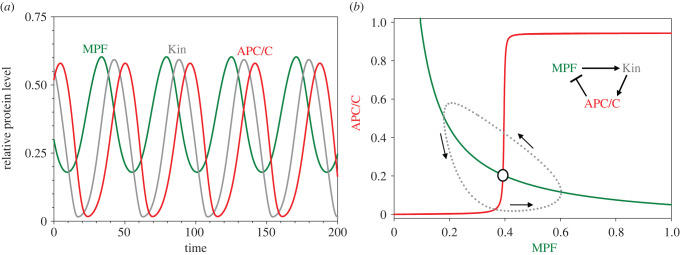
Time-delayed negative feedback loop. (*a*) Time courses of limit cycle oscillation. Parameter values: table 1; *J*_ph,kin_ = *J*_dp,kin_ = 0.01, *J*_ph,apc_ = *J*_dp,apc_ = 0.1, [PP] = [Kin_tot_] = [APC_tot_] = 1. (*b*) Projection of limit cycle oscillation onto a pseudo-phase plane.

In these equations, ‘MPF’ refers to the CycB : Cdk1 dimer (a protein kinase), ‘Kin_P_’ refers to the active (phosphorylated) form of an unspecified ‘intermediary’ protein kinase, ‘APC_P_’ refers to the active form of APC/C, and ‘PP’ denotes a protein phosphatase that opposes the kinase activities. (The intermediary kinase is introduced to provide a time delay in the negative feedback loop between CycB and APC, which is a requirement for limit cycle oscillations; see e.g. [9].) Furthermore, [Kin] = [Kin_tot_] − [Kin_P_] and [APC] = [APC_tot_] − [APC_P_], where [Kin_tot_] and [APC_tot_] are the ‘total’ concentrations, assumed constant. [PP] is also assumed to be a constant concentration. The other parameters: *k*_sy_ and *k*_de_ are rate constants for synthesis and degradation (of cyclin B, the limiting component of MPF), *k*_ph_ and *k*_dp_ are rate constants for substrate phosphorylation and dephosphorylation, and *J*_ph_, and *J*_dp_ are Michaelis constants for the kinases and phosphatases. (The reason for the unexpected ‘gwl’ and ‘dp1’ notation in equation (3.1*b*) will become apparent soon.) Figure 2*a* plots a representative oscillation for ODEs (3.1*a–c*), using the parameter values in table 1. (Computer codes for all the models can be found in the supplementary material.) These oscillations (period approx. 46 min) compare favourably with MPF oscillations in the early frog embryo (period approx. 30 min). In figure 2*b*, we project the limit cycle oscillation onto the plane spanned by [MPF] and [APC_P_]. The red and green curves are ‘nullclines’, calculated by solving equation (3.1*b*) for the pseudo-steady state value of [Kin_P_] as a function of [Kin_T_] and [MPF], substituting this function into equation (3.1*c*), and then treating equation (3.1*a*) and (3.1*c*) as a pair of nonlinear ODEs for [CycB] and [APC_P_]. The green curve is a true nullcline for MPF because the vector field is vertical along this curve. The red curve is a pseudo-nullcline for APC_P_—the orbit (the grey curve) does not cross the ‘nullcline’ curve horizontally—because Kin_P_ induces a time delay between CycB and changes in APC_P_.

**Table 1.** Parameter values for all simulations. For all models, [PP] = [Gwl_tot_] = [APC_tot_] = [Cdc20_tot_] = [B55_tot_] = 1, [ENSA_tot_] = 4; unless otherwise stated, [Cdc20_tot_] = [Wee1_tot_] = [Cdc25_tot_] = 1.

parameter	Model no.			
	1	2	3	4
*k*_sy,cycb_	0.04	0.04	0.04	0.04
*k*_de1,cycb_	0.02	0.02	0.02	0.02
*k*_de2,cycb_	0.4	2	0.5	1
*k*_ph,gwl_	0.2	0.1	2	2
*k*_dp1,gwl_	0.08	0.2	2	0.5
*k*_ph,apc_	0.2	1	1	1
*k*_dp,apc_	0.08	20	20	20
*k*_ph,ensa_		0.2	1	1
*k*_cat_		0.1	1	1
*k*_as1_		10	10	10
*k*_di1_		0.1	0.1	0.1
*k*_dp2,gwl_			20	20
*k*_ph1,cdk_				0.02
*k*_ph2,cdk_				2
*k*_dp1,cdk_				0.2
*k*_dp2,cdk_				2

At the time, this model was appropriate because experimental observations clearly showed a delay between activation of CycB : Cdk1 kinase and CycB degradation (induced by APC/C ubiquitination of CycB) [6]. Goldbeter's assumption that the activations of Kin and APC are ‘ultrasensitive’ response functions was confirmed, in part, by Yang & Ferrell [10], who showed that APC activity as a function of [CycB : Cdk1] is a very steep, sigmoidal function (effective Hill exponent = 32). Nonetheless, the interpretation of these oscillations was unclear because the molecular mechanism of the delay (i.e. the ‘intermediary’ enzyme) was unknown.

### (b) Model no. 2

**Figure 3.**
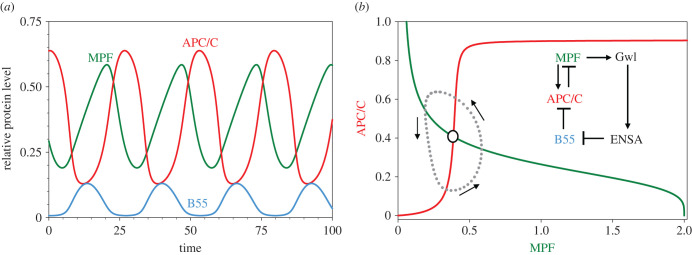
B55-ENSA-Gwl (BEG) pathway (see inset in (*b*)). APC/C is activated by a coherent feed-forward loop from MPF directly and indirectly through Gwl, ENSA and B55. In the figures, ‘APC/C’ refers to the active form of the ubiquitin ligase, namely APC_P_ : Cdc20. (*a*) Time courses of limit cycle oscillation. Parameter values: table 1; *K*_m_ = 0.0008, [dUb]= 0.75. (*b*) Projection of limit cycle oscillation (dotted curve) onto a pseudo-phase plane.

More recently the molecular basis of this delay has become clearer, thanks to a study by Mochida *et al*. [11] and Gharbi-Ayashi *et al*. [12] of the protein phosphatase 2A (PP2A) and its regulatory subunit, B55. These authors found that PP2A:B55 is indirectly inhibited by CycB : Cdk1 during mitosis, by the mechanism illustrated in the inset to figure 3*b*. CycB : Cdk1 phosphorylates and activates a protein kinase called ‘Greatwall’ (Gwl) [13], which in turn phosphorylates and activates ENSA, an inhibitory substrate of PP2A : B55. To connect this pathway to APC/C dynamics, Zhang *et al*. [14] proposed that PP2A : B55 is at least partly responsible for dephosphorylation/inactivation APC/C, as in figure 3*b* (inset). This interaction creates a coherent feed-forward loop: MPF → APC/C, and MPF → Gwl → ENSA ─|B55 ─|APC/C. A physiological advantage of this coherent feed-forward loop is that high kinase activity tends to depress phosphatase activity, and *vice versa*; thereby limiting ‘futile cycling’ of ATP. We model this network by the following ODEs, adapted from Zhang *et al*. [14]:

3.2a $$\begin{array}{rl} \frac{d[\mathrm{MPF}]\;}{dt} & =k_{\mathrm{sy},\mathrm{cycb}}-(k_{\mathrm{de}1,\mathrm{cycb}}+f\;k_{\mathrm{de}2,\mathrm{cycb}})\\ & \;\cdot [\mathrm{MPF}], \end{array}$$

3.2b $$\;\begin{array}{rl} \frac{d[{\mathrm{Gwl}}_{P}]}{dt} & =k_{\mathrm{ph},\mathrm{gwl}}[\mathrm{MPF}]\;[\mathrm{Gwl}]\;\\ & \;-k_{\mathrm{dp}1,\mathrm{gwl}}[\mathrm{PP}]\;[{\mathrm{Gwl}}_{P}], \end{array}$$

3.2c $$\;\begin{array}{rl} \frac{d[{\mathrm{ENSA}}_{P}]}{dt} & =k_{\mathrm{ph},\mathrm{ensa}}[{\mathrm{Gwl}}_{P}][\mathrm{ENSA}]\\ & \;-k_{\mathrm{cat}}[{\mathrm{ENSA}}_{P}B55], \end{array}$$

3.2d $$\;\begin{array}{rl} \frac{d[{\mathrm{APC}}_{P}]}{dt} & =k_{\mathrm{ph},\mathrm{apc}}[\mathrm{MPF}]\;[\mathrm{APC}]\;\\ & \;-k_{\mathrm{dp},\mathrm{apc}}[B55]\;[{\mathrm{APC}}_{P}], \end{array}$$

3.2e $$\begin{array}{rl} \frac{d[{\mathrm{APC}}_{P}C20]}{dt} & =k_{\mathrm{as}1}([{\mathrm{APC}}_{P}]-[{\mathrm{APC}}_{P}C20])([C20_{\mathrm{tot}}]\\ & \;-[{\mathrm{APC}}_{P}C20])-k_{\mathrm{di}1}[{\mathrm{APC}}_{P}C20] \end{array}\;$$

3.2f $$\mathrm{and}\;[{\mathrm{ENSA}}_{P}B55]=\frac{2[{\mathrm{ENSA}}_{P}]\;[B55_{\mathrm{tot}}]}{B+\sqrt{B^{2}-4[{\mathrm{ENSA}}_{P}]\;[B55_{\mathrm{tot}}]}},$$

where $B=[{\mathrm{ENSA}}_{P}+B55_{\mathrm{tot}}]+K_{m}\;,\;K_{m}=(k_{\mathrm{di}2}+k_{\mathrm{cat}})/k_{\mathrm{as}2},$

3.2g $$\begin{array}{rl} {}[{\mathrm{Gwl}}_{\mathrm{tot}}] & =[\mathrm{Gwl}]\;+[{\mathrm{Gwl}}_{P}],\;[{\mathrm{APC}}_{\mathrm{tot}}]=[\mathrm{APC}]\;+[{\mathrm{APC}}_{P}],\\ {}[B55_{\mathrm{tot}}] & =[B55]\;+[{\mathrm{ENSA}}_{P}B55],\\ {}[{\mathrm{ENSA}}_{\mathrm{tot}}] & =[\mathrm{ENSA}]\;+[{\mathrm{ENSA}}_{P,\mathrm{free}}]+[{\mathrm{ENSA}}_{P}B55]\;\\ & =[\mathrm{ENSA}]\;+[{\mathrm{ENSA}}_{P}]\\ \mathrm{and}\;\;\;f & =\frac{\left[{\mathrm{CycBUb}}_{4}\right]}{\left[\mathrm{MPF}\right]}={\left(\frac{\left[{\mathrm{APC}}_{P}C20\right]}{[\mathrm{deUB}]\;}\right)}^{4}\left(1-\frac{\left[{\mathrm{APC}}_{P}C20\right]}{[\mathrm{deUB}]\;}\right)\\ & \;\times {\left(1-{\left(\frac{\left[{\mathrm{APC}}_{P}C20\right]}{[\mathrm{deUB}]\;}\right)}^{5}\right)}^{-1}. \end{array}\}$$

The derivation of these equations requires some explanation. (3.2*a*) [MPF] = [CycB : Cdk1], where Cdk1 is in excess, and CycB is synthesized at constant rate and degraded slowly (*k*_de1_) by a constitutive protease, or rapidly (*k*_de2_) after CycB has been multiply ubiquitinated by APC_P_ : Cdc20; *f* = fraction of CycB pool that is polyubiquitinated, according to an ordered-distributive mechanism [15] with [deUb] = constant activity of a deubiquitinating enzyme. (To be definite, we assume that CycB must be ubiquitinated four times before it is degraded by proteasomes.) (3.2*b*) Gwl is phosphorylated by MPF and dephosphorylated by a constitutive phosphatase, PP. (3.2*c*) ENSA is phosphorylated by Gwl and dephosphorylated by PP2A : B55, abbreviated as ‘B55’ [16]; [ENSA_P_B55] is the concentration of the enzyme–substrate complex, and *k*_as2_, *k*_di2_ and *k*_cat_ are the rate constants for association, dissociation and catalysis of the enzyme–substrate complex. (3.2*d*) APC is phosphorylated by MPF and dephosphorylated by B55. (3.2*e*) APC_P_ binds reversibly with Cdc20 to form the active ubiquitin ligase, APC_P_ : Cdc20; the association and dissociation rate constants are *k*_as1_ and *k*_di1_. (3.2*f*) The enzyme–substrate complex [ENSA_P_B55] is calculated by the ‘total quasi-steady state approximation’ [17]. Notice that [ENSA_P_] = [ENSA_P,free_] + [ENSA_P_B55].

In figure 3, we plot oscillations of [MPF], [B55] and [APC_P_C20] predicted by this model as functions of time (*a*) and as a limit cycle projected on the plane spanned by [MPF] and [APC_P_C20] (*b*). The oscillations are ‘soft’ (nearly sinusoidal), and the phosphatase (B55) is only about 90° out-of-phase with the kinase (MPF), so ‘futile cycling’ is reduced but not as much as would be possible with phase shift of a 180°.

In figure 3*b*, we also plot pseudo-nullclines for MPF (green) and APC_P_C20 (red). The pseudo-nullclines are calculated using the capacity of XPPAUT to plot one-parameter bifurcation diagrams for a reduced set of ODEs [18]; XPPAUT is freely available from http://www.math.pitt.edu/~bard/xpp/xpp.html. For example, to calculate the pseudo-nullcline for MPF, we remove equation (3.2*e*) from the ODEs, treating [APC_P_C20] as a parameter, and plot the one-parameter bifurcation diagram for the remaining equations, with [MPF] as the variable and [APC_P_C20] as the parameter. Similarly, for the APC_P_C20 nullcline, we remove equation (3.2*a*) from the ODEs, treating [MPF] as a parameter, and plot the one-parameter bifurcation diagram for the remaining equations, with [APC_P_C20] as the variable and [MPF] as the parameter. Because our dynamical system is five-dimensional, the projected orbit (the dotted line in figure 3*b*) does not cross the pseudo-nullclines horizontally and vertically, as it would for true nullclines of a two-dimensional dynamical system; but, nonetheless, the pseudo-nullclines provide a useful projection of the vector field of the full dynamical system, and we shall use pseudo-nullclines, calculated in this way, to describe more complex mechanisms to come.

### (c) Model no. 3

**Figure 4.**
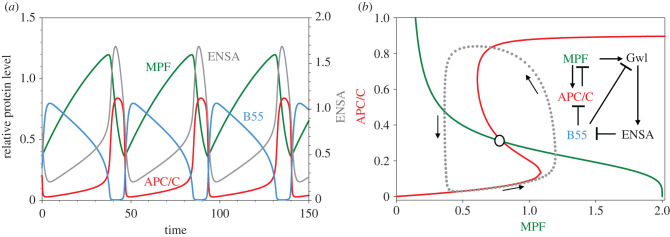
APC is regulated by a bistable switch. Inset to (*b*) shows the reaction network. As before, ‘APC/C’ is the variable [APC_P_C20]. (*a*) Time courses of limit cycle oscillation. MPF, B55 and APC/C are plotted with respect to the left axis and ENSA with respect to the right axis. Parameter values: table 1; *K*_m_ = 0.0026, [dUb] = 0.5. (*b*) Projection of limit cycle oscillation onto a pseudo-phase plane. Notice that APC/C activity is now a bistable function of MPF.

Another level of control was proposed by Vinod & Novak [19] and experimentally confirmed by Mochida *et al*. [20] that PP2A : B55 dephosphorylates Gwl (see inset to figure 4*b*). They modelled the revised diagram by the dynamical system (3.2) with equation (3.2*b*) replaced by

3.3b $$\begin{array}{c} \frac{d[{\mathrm{Gwl}}_{P}]}{dt}=k_{\mathrm{ph},\mathrm{gwl}}[\mathrm{MPF}]\;[\mathrm{Gwl}]\;\\ \;-\left(k_{\mathrm{dp}1,\mathrm{gwl}}[\mathrm{PP}]+k_{\mathrm{dp}2,\mathrm{gwl}}[B55]\right)\cdot [{\mathrm{Gwl}}_{P}].\; \end{array}$$

As before, we plot the time courses and pseudo-phase plane in figure 4*a*,*b*. This seemingly slight modification to the model causes a dramatic qualitative change to the phase plane. The APC_P_ pseudo-nullcline is now S-shaped, i.e. APC/C activity is now a bistable function of MPF because PP2A : B55 activity is now controlled by a bistable switch. Bistability of B55 derives from the positive feedback loop: Gwl → ENSA ─| B55 ─|Gwl, which is modulated by MPF. Bistability of B55 drives bistability of APC/C. That APC/C activity is a bistable function of MPF was already a reasonable hypothesis, given the very abrupt activation of APC/C by increasing activity of MPF (*n*_H_ = 32, observed by [10]), and, indeed, it has been confirmed recently by Kamenz *et al*. [21].

As is evident in figure 4, the oscillations of Model no. 3 are now ‘hard’ (i.e. abrupt jumps between B55 active, APC/C inactive and *vice versa*). Now MPF activity looks more like the observed mitotic cycles of early embryos, with a steady rise of [MPF] and an abrupt fall. But futile cycling is still a problem because MPF and B55 are both active during much of interphase.

## 4. Cell cycle oscillations in frog egg extracts

**Figure 5.**
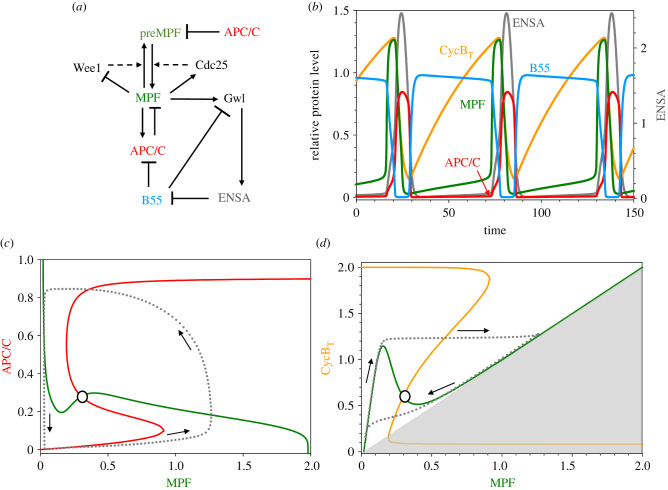
APC and MPF are both regulated by bistable switches. (*a*) The reaction network. MPF + preMPF = CycB_T_. (*b*) Time courses of limit cycle oscillation. As before, ‘APC/C’ is the variable [APC_P_C20]. MPF, B55, CycB_T_ and APC/C are plotted with respect to the left axis and ENSA with respect to the right axis. Parameter values: table 1; *K*_m_ = 0.0026, [dUb] = 0.5, [CAP] = 0.3. (*c*) Pseudo-phase plane, [APC_P_C20] versus [MPF]. Dashed line shows projection of limit cycle oscillation in (*b*). Notice that MPF activity is now a bistable function of APC/C. (*d*) Pseudo-phase plane, [CycB_T_] versus [MPF]. Dashed line shows projection of limit cycle oscillation in (*b*). Grey region, [MPF] > [CycB_T_], is ‘unreachable’.

In 1989, Murray & Kirschner [22] described how to prepare frog egg extracts that exhibit sustained oscillations of MPF activity *in vitro*. Unlike *in vivo*, the *in vitro* oscillations are marked by significant inhibitory Cdk1-phosphorylation in interphase. As worked out by Nurse & Hayles [23] in fission yeast, Cdk1 is inactivated by phosphorylation on a tyrosine residue by Wee1 kinase (CycB : Cdk1 → CycB : Cdk1_P_) and reactivated by dephosphorylation by Cdc25 phosphatase. Furthermore, Wee1 is inactivated by phosphorylation by MPF, and Cdc25 is activated by phosphorylation by MPF [5]. It is known that Wee1 and Cdc25 are phosphorylated on multiple sites by MPF, and that the activity of each is a sigmoid function of MPF activity [24,25]. This reaction network is illustrated in figure 5*a*.

### (a) Model no. 4

Introducing these reactions in Model no. 3, we obtain

4.1a $$\frac{d[{\mathrm{CycB}}_{T}]}{dt}=k_{\mathrm{sy},\mathrm{cycb}}-\left(k_{\mathrm{de}1,\mathrm{cycb}}+f\;k_{\mathrm{de}2,\mathrm{cycb}}\right)\cdot [{\mathrm{CycB}}_{T}],$$

4.1b d[MPF] dt=ksy,cycb−(kde1,cycb+f kde2,cycb)⋅[MPF] −(kph1,cdk[Wee1P]+kph2,cdk[Wee1])⋅[MPF] +(kdp1,cdk[Cdc25] +kdp2,cdk[Cdc25P])×([CycBT]−[MPF]).

d[Gwl_P_]/d*t* is given by equation (3.3*b*), d[ENSA_P_]/d*t*, d[APC_P_]/d*t* and d[APC_P_C20]/d*t* are given by equations (3.2*c*–*e*), [ENSA_P_B55] by (3.2*f*), and B through *f* by (3.2*g*). Also

4.1c $$\frac{[\text{Wee1}]\;}{[\mathrm{Wee}1_{\mathrm{tot}}]}=\left(1-{\left(\frac{\left[\mathrm{MPF}\right]}{[\mathrm{CAP}]\;}\right)}^{5}\right){\left(1-{\left(\frac{\left[\mathrm{MPF}\right]}{[\mathrm{CAP}]\;}\right)}^{9}\right)}^{-1}\;$$

4.1d $$\begin{array}{rl} \;\mathrm{and}\;\frac{[\mathrm{Cdc}25_{P}]}{[\mathrm{Cdc}25_{\mathrm{tot}}]} & ={\left(\frac{\left[\mathrm{MPF}\right]}{[\mathrm{CAP}]\;}\right)}^{5}\left(1-{\left(\frac{\left[\mathrm{MPF}\right]}{[\mathrm{CAP}]\;}\right)}^{4}\right)\\ & \;\times {\left(1-{\left(\frac{\left[\mathrm{MPF}\right]}{[\mathrm{CAP}]\;}\right)}^{9}\right)}^{-1}, \end{array}$$

4.1e $$\begin{array}{rl} \mathrm{where}\;[\mathrm{Wee}1_{P}] & =[\mathrm{Wee}1_{\mathrm{tot}}]-[\text{Wee1}]\\ \mathrm{and}\;[\mathrm{Cdc}25] & =[\mathrm{Cdc}25_{\mathrm{tot}}]-[\mathrm{Cdc}25_{P}]. \end{array}\}$$

These ODEs require some explanation. (4.1*a*) [CycB_T_] = [CycB : Cdk1] + [CycB : Cdk1_P_] = ‘total cyclin B’. (4.1*b*) [MPF] = [CycB : Cdk1] = ‘active MPF’. (4.1*c*,*d*) [Wee1] = concentration of the ‘more active’ (i.e. unphosphorylated) form of Wee1, and [Cdc25_P_] = concentration of the ‘more active’ (i.e. phosphorylated) form of Cdc25. In this model, Wee1 and Cdc25 are phosphorylated on eight sites by MPF by an ordered-distributive mechanism [15], with counter-acting phosphatase ‘CAP’, assuming that Wee1_P5–8_ are less active and Cdc25_P5–8_ are more active.

The time courses of selected components of this model are plotted in figure 5*b*, along with two pseudo-phase plane portraits in figure 5*c*,*d*. The time courses of CycB_T_ and MPF agree nicely with the measurements of Pomerening *et al*. [26] on frog egg extracts, if 1 time unit is approximately equal to 1 min. Comparing the phase plane portraits in figures 4*b* and 5*c*, we see that APC/C activity is still a bistable function of [MPF] (red curves in figures 4*b* and 5*c*), but now MPF activity is a bistable function of [APC_P_C20] (green curve in figure 5*c*). The bistability of MPF activity is better visualized in the MPF-CycB_T_ phase plane (green curve in figure 5*d*). As [CycB_T_] increases, MPF activity is low at first, but jumps abruptly to high activity ([MPF] approximately equal to [CycB_T_]) for [CycB_T_] greater than 1.2. Then, as [CycB_T_] decreases (as cyclin B is degraded by active APC/C), MPF activity stays high until [CycB_T_] drops below 0.5. (This bistability of MPF activity for intermediate levels of [CycB_T_] was demonstrated experimentally in frog egg extracts by Sha *et al*. [27] and Pomerening *et al*. [28].) Notice, in figure 5*d*, that [CycB_T_] continues to decrease even after MPF is inactivated by tyrosine-phosphorylation, because there is a time delay between the inactivation of MPF and the subsequent inactivation of APC/C by PP2A:B55.

Dual bistability in Model no. 4 has the added advantage that MPF and B55 activities are now strictly out-of-phase. Because the kinase and phosphatase are not active at the same time, mitotic proteins are highly phosphorylated in mitosis and dephosphorylated in interphase, with minimal futile cycling.

Another potential modification worth mentioning is the likely possibility that PP2A : B55 is one of the phosphatases that activates Wee1 and inactivates Cdc25_P_ [29]. This interaction creates two ‘interlinked’ bistable switches [20,30] that are coupled by mutual inhibition (MPF → Gwl ─|B55 and B55 → Wee1 ─| MPF). As Rata *et al*. [30] have shown, this network can exhibit three coexisting stable steady states: (1) interphase, with low MPF activity and high B55 activity, (2) metaphase, with high MPF and low B55, and (3) prophase, with intermediate activities of MPF and B55. We will not comment further on this behaviour, because it has yet to be carefully studied theoretically or convincingly demonstrated experimentally [30].

## 5. Checkpoints

Instead, using Model no. 4, we turn our attention to the three checkpoints that monitor genome integrity during the cell division cycle: the G1 checkpoint, the G2 checkpoint and the spindle assembly checkpoint (SAC). (In figure 1, they are denoted Q1, Q2 and Q3, respectively.) We describe these checkpoints in terms of pseudo-phase plane portraits. For Model no. 4, as illustrated in figure 5*d*, the (pseudo) nullclines are N-shaped (green) and Z-shaped (orange), and they intersect on the intermediate (unstable) branches of both nullclines; hence, the dynamical system for this particular choice of kinetic parameter values executes sustained limit cycle oscillations (the dotted curve). Clearly, however, as parameter values change, the nullclines will move relative to each other, and new (stable) steady states may be created. Three such configurations are relevant to cell cycle checkpoints: (1) a stable steady state at low [CycB_T_] and low [MPF]—that would be a cell arrested in G1; (2) a stable steady state at high [CycB_T_] and low [MPF]—a cell primed to enter mitosis, but not yet committed to do so; and (3) a stable steady state of high [CycB_T_] and high [MPF]—a cell arrested in metaphase, with high MPF activity (until all chromosomes are properly aligned on the mitotic spindle).

**Figure 6.**
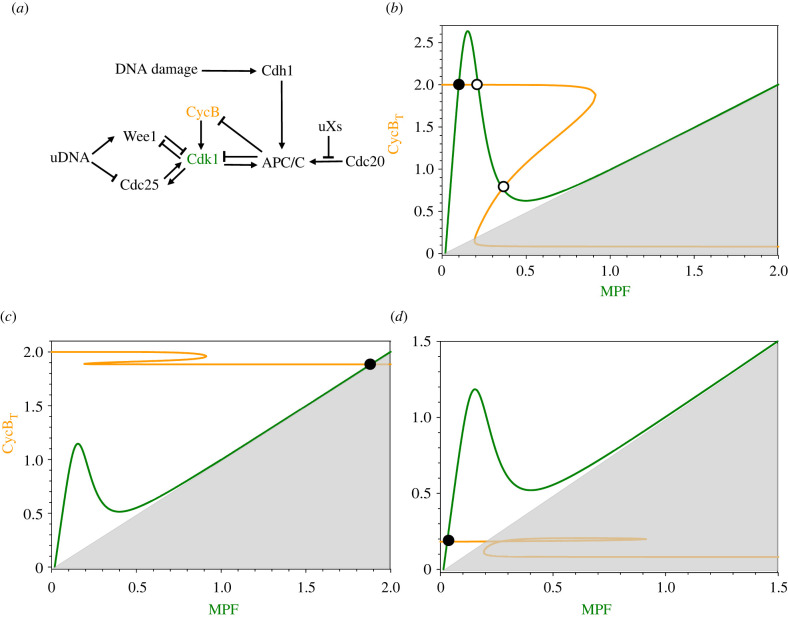
Checkpoint signalling. (*a*) The reaction network. uDNA, unreplicated DNA; uXs, unaligned chromosomes; Cdc20 and Cdh1, alternative targeting subunits of APC/C. (*b*) G2 checkpoint. Unreplicated DNA raises the peak of the N-shaped, MPF nullcline (green curve) so that it crosses the Z-shaped, CycB_T_ nullcline (orange curve) at a stable steady state (filled circle) of high [CycB_T_] and low MPF activity (G2 arrest). Parameter values: table 1, Model no. 4; [Cdc25_tot_] = 0.4. (*c*) Spindle assembly checkpoint. Unaligned chromosomes inhibit Cdc20, thereby blocking APC/C-mediated degradation of polyubiquitinated CycB and creating a stable steady state (filled circle) of high [CycB_T_] and high MPF activity (mitotic arrest). Parameter values: table 1, Model no. 4; [Cdc20_tot_] = 0.1. (*d*) G1 checkpoint. Damaged DNA stabilizes Cdh1, the subunit that targets APC/C activity to cyclins in G1 phase. Hence, cyclins A and B cannot accumulate, and the cell is blocked at a stable steady state (filled circle) of low [CycB_T_] and low MPF and SPF activities (G1 arrest). Parameter values: table 1, Model no. 4; *k*_de3,cycb_ = 0.2.

To model these checkpoints, we extend Model no. 4 to include surveillance signals from DNA damage, unreplicated DNA and unaligned chromosomes, as in figure 6*a*.

### (a) Model no. 4—G2

To illustrate a G2 checkpoint (figure 6*b*), we assume that unreplicated DNA induces sequestration of approximately 40% of Cdc25 in the cytoplasm (we decrease [Cdc25_tot_] to 0.4). This parameter change creates a pair of steady states—a node and a saddle point (filled circle and open circle)—on the upper branch of the CycB_T_ nullcline, thereby blocking progression into M phase. As DNA replication is completed, [Cdc25_tot_] increases back to 1, and the limit cycle in figure 5*d* is reestablished by a SNIC bifurcation (saddle-node on an invariant circle).

### (b) Model no. 4—spindle assembly checkpoint

Unaligned chromosomes induce the SAC by generating ‘mitotic checkpoint complexes' (MCCs) that sequester Cdc20, preventing it from binding to APC_P_. To model this mechanism, we decrease the total amount of Cdc20 available for binding to APC_P_ (we decrease [Cdc20_tot_] from 1 to 0.2). This parameter change creates a stable state (filled circle) of ‘mitotic arrest’ on the right branch of the MPF nullcline (high MPF activity, figure 6*c*). When all chromosomes are properly aligned on the mitotic spindle, MCC is rapidly disassembled, the full complement of Cdc20 becomes available to APC_P_, and the limit cycle in figure 5*d* is recreated (again by a SNIC bifurcation).

### (c) Model no. 4—G1

During G1 phase, when Cdc20 is absent, APC/C binds to Cdh1 (a protein homologous to Cdc20) and induces polyubiquitinylation of cyclins, until the start of DNA replication [31]. Unlike Cdc20, Cdh1 binds to unphosphorylated APC/C. To model the role of Cdh1, we rewrite equation (4.1*a*) as

5.1 $$\begin{array}{cc} \frac{d[\mathrm{Cyc}B_{T}]}{dt}\; & =k_{\mathrm{sy},\mathrm{cycb}}-(k_{\mathrm{de}1,\mathrm{cycb}}+f\;k_{\mathrm{de}2,\mathrm{cycb}}\;\\ \; & +k_{\mathrm{de}3,\mathrm{cycb}}([\mathrm{AP}C_{\mathrm{tot}}]-[\mathrm{AP}C_{P}]))[\mathrm{Cyc}B_{T}].\; \end{array}$$

In figure 6*d*, we set *k*_de3,cycb_ = 0.2. The activity of Cdh1 : APC/C keeps CycBT very low (at filled circle), so the cell is arrested in G1 phase. To lift the checkpoint, Cdh1 needs to be inhibited by phosphorylation by G1-active CDKs, which only happens in the absence of DNA damage [31].

## 6. Size control of cell division in fission yeast

So far, we have focused our attention on limit cycle oscillations of MPF in early embryonic divisions of a fertilized frog egg and in frog egg extracts, because these preparations illustrate the basic principles of mitotic control by CDKs and their opposing phosphatases. This approach highlights the time-keeping aspects of cell cycle regulation in embryos, where periodic alternations between interphase and mitosis are governed by limit cycle oscillations. Then, we touched briefly on mechanisms of checkpoint regulation—only enough to show that the decision-making functions of checkpoints are implemented by bifurcations (figure 6) that introduce a stable steady state into the orbit of the limit cycle. In technical terms, these are saddle-node-on-invariant circle, or ‘SNIC,’ bifurcations. In this section, we illustrate how the G2-SNIC bifurcation plays a major role in understanding ‘size-control’ of the cell division cycle in fission yeast (*Schizosaccharomyces pombe*). Similar considerations apply to budding yeast, green algae and animal cells, for which we refer readers to literature sources [32–35].

Progression through the cell division cycle of fission yeast is strongly coupled to cell growth, as has been demonstrated in many experiments over the years [36]. Similarly convincing experimental evidence demonstrates the role of size control in other simple eukaryotic organisms [37], like budding yeast [38,39] and slime moulds [40–42], and there is some evidence for size control of the division of mammalian cells in culture [43], although less conclusive. In wild-type fission yeast cells, cell size governs progression through the cell cycle at the G2 checkpoint [36], and it seems to work by a cell-size-dependent increase in the concentration of Cdc25 during G2 phase [44]. We introduce this effect into Model no. 4 by making [Cdc25_tot_] a function of cell ‘size’, *V*(*t*), which (we assume) increases exponentially from birth to division and is reduced by a factor of 2 at cell division; i.e. we append to Model no. 4

6.1 $$\begin{array}{c} {}[\mathrm{Cdc}25_{\mathrm{tot}}]=V(t),\;\;\frac{dV}{dt}=\mu V,\;\\ V(t)\to V(t)/2\;\\ \mathrm{at}\;\mathrm{cell}\;\mathrm{division}.\; \end{array}\}$$

The cell divides if it first enters mitosis (i.e. [MPF] increases above a predefined threshold) and then exits mitosis ([MPF] decreases below a different threshold).

**Figure 7.**
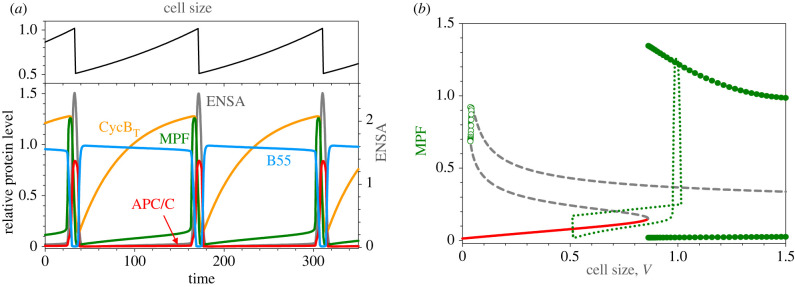
Size-controlled mitotic cycle in fission yeast. (*a*) Upper panel: time course of cell size, *V*(*t*), which increases exponentially (*μ* = 0.005 min^−1^, mass doubling time = 140 min) and drops by a factor of 2 at cell division (when [MPF] drops below 0.2). Lower panel: time courses of representative variables. All the variables are read off the left axis, except for ENSA, which is read off the right axis. (*b*) A one-parameter bifurcation diagram, for [MPF] as a function of *V*, assuming *V* is constant in the nonlinear ODEs. Solid red curve, stable steady states (nodes); intermediate dashed grey curve, unstable saddle points; upper dashed grey curve, unstable nodes; open green circles, unstable limit cycles emanating from a Hopf bifurcation at *V* ≈ 0.05; filled green circles, maxima and minima of stable limit cycle oscillations emanating from a SNIC bifurcation at *V* ≈ 0.85.

Implementing this model, we show in figure 7*a* the time course of oscillations. Cell size, *V*(*t*), increases exponentially and drops twofold at cell division. Notice that the phosphatase, PP2A : B55, is active during most of interphase (short G1 + S phase + long G2, as in wild-type fission yeast cells). Total cyclin B accumulates steadily in interphase, but MPF activity is very low, because Cdk1 is phosphorylated by Wee1. At the end of G2 phase, MPF is rapidly activated by Cdc25 phosphatase activity, and the cell enters mitosis. Meanwhile, B55 is inactivated by the MPF–Gwl–ENSA pathway, and, consequently, APC is phosphorylated/activated by MPF. APC_P_ binds with Cdc20 and drives degradation of CycB, and falling activity of MPF triggers cell division. Newborn daughter cells repeat the process. Notice that the cell cycle time (G1 + S + G2 + M) is exactly equal to the mass doubling time.

In figure 7*b*, we portray the ‘logic’ of these growth-controlled cell division cycles using the concept of a one-parameter bifurcation diagram from the theory of dynamical systems. This diagram depicts the characteristic states of a dynamical system in dependence on the value of a parameter in the differential equations. In this case, the ‘state’ of the system is represented by the value of the dynamical variable [MPF], and the ‘parameter’ is cell size *V*. We can treat cell size as a parameter because the rate of change of *V*, namely *μV* (with *μ* = 0.005) is very slow compared with the rates of change of the dynamical variables [CycB_T_], [MPF], [APC_P_], etc. For this reason, we may set *V* constant and solve the remaining ODEs for the behaviour of the underlying molecular control system at that particular size. In the one-parameter bifurcation diagram, we see that, at small size, the only stable solution of the ODEs is a steady state (red line) of low MPF. As the cell slowly grows, the dynamical system will cling to this stable steady state at first. Let us pick up this ‘cell cycle trajectory’ (the green dotted curve in figure 7*b*) at *V* ≈ 0.5. The cell continues to grow, at low MPF, until the trajectory reaches the SNIC bifurcation at *V* ≈ 0.85. At this bifurcation point, the stable steady state of low MPF (a ‘node’) coalesces with the unstable steady state (on the intermediate locus—the grey dashed curve—of ‘saddle points’), giving rise to a stable limit cycle (an ‘invariant circle’) whose maximum and minimum excursions of MPF activity are plotted by the green filled circles. Upon passing the SNIC bifurcation point, the growing cell should ‘hop’ onto the limit cycle oscillation, but the speed of the transition is very slow at the bifurcation point. Hence, the cell continues to grow (slowly) until it passes some distance from the SNIC point (*V* ≈ 1) before the cell cycle trajectory is captured by the limit cycle. As MPF activity increases abruptly, the cell is driven into mitosis, and as MPF activity drops rapidly, as active APC/C degrades CycB, the cell exits mitosis and divides. As the cell divides, one can see the cell cycle trajectory drop abruptly from *V* ≈ 1 to *V* ≈ 0.5. At this size, the only long-term behaviour of the dynamical system is the stable steady state of low MPF. The newborn cell enters G1 phase, which (in fission yeast) is very short. Soon thereafter, the cell has enough CDK activity to enter S phase; even the residual kinase activity of tyrosine-phosphorylated MPF is enough to initiate DNA replication [45]. The cell will not enter mitosis until MPF is fully activated, as just described.

Notice that this description of the cell cycle trajectory is independent of the specific growth rate *μ* of the cells (within a broad range), so, by this mechanism, the interdivision time will always equal the mass doubling time, a characteristic feature of unicellular eukaryotic organisms.

## 7. Discussion

Living cells exhibit many types of oscillatory behaviours, such as circadian rhythms, Ca^2+^ and cyclic AMP oscillations, pulsatile hormone secretion, periodic somitogenesis and oscillatory expression of transcription factors like p53 and NF-κB [46]. In each of these cases, oscillations are central to the time-keeping function of the process. But the cell cycle is not intrinsically a periodic process. The primary function of the cell cycle is to accurately replicate the cell's genome and to partition the replicated chromosomes evenly between the two daughter cells. Hence, the cell cycle is monitored by surveillance mechanisms that can block further progression if problems arise, such as DNA damage, incomplete DNA replication or aberrant alignment of chromosomes on the mitotic spindle. So, progression through the cell cycle is, in most circumstances, more like a copy machine (which can be halted by a paper jam or an empty toner cartridge) than like an alarm clock (which continues to tick whether one gets out of bed or not).

Nonetheless, there are specific circumstances in which cell division cycles proceed with clock-like regularity. For example, in laboratory settings, when cells are cultured in rich growth media and protected from genome hazards, they grow and divide with a roughly constant interdivision time. A more natural example is that of the early mitotic cycles of a fertilized egg, like frog and sea urchin eggs. These division cycles are typically rapid and synchronous (up to the mid-blastula transition), and they often proceed without safeguarding checkpoint mechanisms.

The mitotic cycles of a fertilized egg are rather like the copy machine in the departmental office during exam week, when it is outputting exam copies at top speed; or like the clothes washing machine in a home with a new baby, which is dealing with dirty linen non-stop. Under less stressful conditions, these machines work at a more leisurely pace, dealing with demands as they arise and heeding checkpoint signals with equanimity. We might say that the free-running mitotic cycles of early embryos, as central as they were to unravelling the biochemistry of cell cycle control in eukaryotes and as appealing as they are to describing the dynamical properties of cell cycle progression, are rather ‘special cases’ of cell cycle control. Much more representative are the unhurried replication–division cycles of growing yeast cells and the somatic cells of plants and animals. In these common circumstances *in vivo* (rather than in laboratory cultures), cell cycle progression is less like a ‘clock’ (a limit cycle) and more like a ‘copy machine,’ i.e. a sequence of copy-and-collate functions governed by external demands (driven by autonomous cell growth and/or responding to specific growth factors) and internal requirements (completion of DNA synthesis, repair of any DNA damage, perfect alignment of sister chromatids on the mitotic spindle). These physiological constraints make the study of cell cycle control a delightfully complex example of time-keeping and decision-making in living cells.

## Data Availability

Computer programs for simulating the models described in the paper are provided in the electronic supplementary material.

## References

[1] Morgan DO. 2007 The cell cycle: principles of control. London, UK: New Science Press.

[2] Murray AW, Kirschner MW. 1989 Dominoes and clocks: the union of two views of the cell cycle. Science **246**, 614-621. (10.1126/science.2683077) 2683077

[3] Tyson JJ, Novak B. 2008 Temporal organization of the cell cycle. Curr. Biol. **18**, R759-R768. (10.1016/j.cub.2008.07.001) 18786381 PMC2856080

[4] Nurse P. 1990 Universal control mechanism regulating onset of M-phase. Nature **344**, 503-508. (10.1038/344503a0) 2138713

[5] Coleman TR, Dunphy WG. 1994 Cdc2 regulatory factors. Curr. Opin. Cell Biol. **6**, 877-882. (10.1016/0955-0674(94)90060-4) 7880537

[6] Felix MA, Labbe JC, Doree M, Hunt T, Karsenti E. 1990 Triggering of cyclin degradation in interphase extracts of amphibian eggs by cdc2 kinase. Nature **346**, 379-382. (10.1038/346379a0) 2142754

[7] Ferrell Jr JE, Wu M, Gerhart JC, Martin GS. 1991 Cell cycle tyrosine phosphorylation of p34cdc2 and a microtubule-associated protein kinase homolog in *Xenopus* oocytes and eggs. Mol. Cell Biol. **11**, 1965-1971. (10.1128/mcb.11.4.1965-1971.1991) 2005892 PMC359881

[8] Goldbeter A. 1991 A minimal cascade model for the mitotic oscillator involving cyclin and cdc2 kinase. Proc. Natl Acad. Sci. USA **88**, 9107-9111. (10.1073/pnas.88.20.9107) 1833774 PMC52661

[9] Novak B, Tyson JJ. 2008 Design principles of biochemical oscillators. Nat. Rev. Mol. Cell Biol. **9**, 981-991. (10.1038/nrm2530) 18971947 PMC2796343

[10] Yang Q, Ferrell Jr JE. 2013 The Cdk1–APC/C cell cycle oscillator circuit functions as a time-delayed, ultrasensitive switch. Nat. Cell Biol. **15**, 519-525. (10.1038/ncb2737) 23624406 PMC3728279

[11] Mochida S, Maslen SL, Skehel M, Hunt T. 2010 Greatwall phosphorylates an inhibitor of protein phosphatase 2A that is essential for mitosis. Science **330**, 1670-1673. (10.1126/science.1195689) 21164013

[12] Gharbi-Ayachi A, Labbe JC, Burgess A, Vigneron S, Strub JM, Brioudes E, Van-Dorsselaer A, Castro A, Lorca T. 2010 The substrate of Greatwall kinase, Arpp19, controls mitosis by inhibiting protein phosphatase 2A. Science **330**, 1673-1677. (10.1126/science.1197048) 21164014

[13] Yu J, Zhao Y, Li Z, Galas S, Goldberg ML. 2006 Greatwall kinase participates in the Cdc2 autoregulatory loop in *Xenopus* egg extracts. Mol. Cell **22**, 83-91. (10.1016/j.molcel.2006.02.022) 16600872

[14] Zhang T, Tyson JJ, Novak B. 2013 Role for regulated phosphatase activity in generating mitotic oscillations in *Xenopus* cell-free extracts. Proc. Natl Acad. Sci. USA **110**, 20 539-20 544. (10.1073/pnas.1318065110) PMC3870745 24297885

[15] Kapuy O, Barik D, Sananes MR, Tyson JJ, Novak B. 2009 Bistability by multiple phosphorylation of regulatory proteins. Prog. Biophys. Mol. Biol. **100**, 47-56. (10.1016/j.pbiomolbio.2009.06.004) 19523976 PMC2784190

[16] Williams BC, Filter JJ, Blake-Hodek KA, Wadzinski BE, Fuda NJ, Shalloway D, Goldberg ML. 2014 Greatwall-phosphorylated Endosulfine is both an inhibitor and a substrate of PP2A-B55 heterotrimers. eLife **3**, e01695. (10.7554/eLife.01695) 24618897 PMC3949306

[17] Kim JK, Tyson JJ. 2020 Misuse of the Michaelis–Menten rate law for protein interaction networks and its remedy. PLoS Comput. Biol. **16**, e1008258. (10.1371/journal.pcbi.1008258) 33090989 PMC7581366

[18] Ermentrout B. 2002 Simulating, analyzing, and animating dynamical systems: a guide to XPPAUT for researchers and students. Philadelphia, PA: Society for Industrial and Applied Mathematics.

[19] Vinod PK, Novak B. 2015 Model scenarios for switch-like mitotic transitions. FEBS Lett. **589**, 667-671. (10.1016/j.febslet.2015.02.007) 25683003

[20] Mochida S, Rata S, Hino H, Nagai T, Novák B. 2016 Two bistable switches govern M phase entry. Curr. Biol. **26**, 3361-3367. (10.1016/j.cub.2016.10.022) 27889260 PMC5196020

[21] Kamenz J, Gelens L, Ferrell Jr JE. 2021 Bistable, biphasic regulation of PP2A-B55 accounts for the dynamics of mitotic substrate phosphorylation. Curr. Biol. **31**, 794-808.E6. (10.1016/j.cub.2020.11.058) 33357450 PMC7904671

[22] Murray AW, Kirschner MW. 1989 Cyclin synthesis drives the early embryonic cell cycle. Nature **339**, 275-280. (10.1038/339275a0) 2566917

[23] Nurse P, Hayles J. 2019 Using genetics to understand biology. Heredity (Edinb.) **123**, 4-13. (10.1038/s41437-019-0209-z) 31189902 PMC6781147

[24] Kim SY, Ferrell Jr JE. 2007 Substrate competition as a source of ultrasensitivity in the inactivation of Wee1. Cell **128**, 1133-1145. (10.1016/j.cell.2007.01.039) 17382882

[25] Trunnell NB, Poon AC, Kim SY, Ferrell Jr JE. 2011 Ultrasensitivity in the regulation of Cdc25C by Cdk1. Mol. Cell **41**, 263-274. (10.1016/j.molcel.2011.01.012) 21292159 PMC3060667

[26] Pomerening JR, Kim SY, Ferrell Jr JE. 2005 Systems-level dissection of the cell-cycle oscillator: bypassing positive feedback produces damped oscillations. Cell **122**, 565-578. (10.1016/j.cell.2005.06.016) 16122424

[27] Sha W, Moore J, Chen K, Lassaletta AD, Yi CS, Tyson JJ, Sible JC. 2003 Hysteresis drives cell-cycle transitions in *Xenopus laevis* egg extracts. Proc. Natl Acad. Sci. USA **100**, 975-980. (10.1073/pnas.0235349100) 12509509 PMC298711

[28] Pomerening JR, Sontag ED, Ferrell Jr JE. 2003 Building a cell cycle oscillator: hysteresis and bistability in the activation of Cdc2. Nat. Cell Biol. **5**, 346-351. (10.1038/ncb954) 12629549

[29] Zhao Y, Haccard O, Wang R, Yu J, Kuang J, Jessus C, Goldberg ML. 2008 Roles of Greatwall kinase in the regulation of cdc25 phosphatase. Mol. Biol. Cell **19**, 1317-1327. (10.1091/mbc.E07-11-1099) 18199678 PMC2291418

[30] Rata S et al. 2018 Two interlinked bistable switches govern mitotic control in mammalian cells. Curr. Biol. **28**, 3824-3832.E6. (10.1016/j.cub.2018.09.059) 30449668 PMC6287978

[31] Cappell SD, Chung M, Jaimovich A, Spencer SL, Meyer T. 2016 Irreversible APC^Cdh1^ inactivation underlies the point of no return for cell-cycle entry. Cell **166**, 167-180. (10.1016/j.cell.2016.05.077) 27368103 PMC6649667

[32] Battogtokh D, Tyson JJ. 2004 Bifurcation analysis of a model of the budding yeast cell cycle. Chaos **14**, 653-661. (10.1063/1.1780011) 15446975

[33] Heldt FS, Tyson JJ, Cross FR, Novák B. 2020 A single light-responsive sizer can control multiple-fission cycles in *Chlamydomonas*. Curr. Biol. **30**, 634-644.E7. (10.1016/j.cub.2019.12.026) 31928875 PMC11869391

[34] Qu Z, MacLellan WR, Weiss JN. 2003 Dynamics of the cell cycle: checkpoints, sizers, and timers. Biophys. J. **85**, 3600-3611. (10.1016/S0006-3495(03)74778-X) 14645053 PMC1303665

[35] Borisuk MT, Tyson JJ. 1998 Bifurcation analysis of a model of mitotic control in frog eggs. J. Theor. Biol. **195**, 69-85. (10.1006/jtbi.1998.0781) 9802951

[36] Wood E, Nurse P. 2015 Sizing up to divide: mitotic cell-size control in fission yeast. Annu. Rev. Cell Dev. Biol. **31**, 11-29. (10.1146/annurev-cellbio-100814-125601) 26566110

[37] Tyson JJ. 1985 The coordination of cell growth and division — intentional or incidental? Bioessays **2**, 72-77. (10.1002/bies.950020208)

[38] Johnston GC, Pringle JR, Hartwell LH. 1977 Coordination of growth with cell division in the yeast *Saccharomyces cerevisiae*. Exp. Cell Res. **105**, 79-98. (10.1016/0014-4827(77)90154-9) 320023

[39] Schmoller KM, Skotheim JM. 2015 The biosynthetic basis of cell size control. Trends Cell Biol. **25**, 793-802. (10.1016/j.tcb.2015.10.006) 26573465 PMC6773270

[40] Sudbery PE, Grant WD. 1976 The control of mitosis in *Physarum polycephalum*: the effect of delaying mitosis and evidence for the operation of the control mechanism in the absence of growth. J. Cell Sci. **22**, 59-65. (10.1242/jcs.22.1.59) 135767

[41] Sudbery PE, Grant WD. 1975 The control of mitosis in *Physarum polycephalum*. The effect of lowering the DNA : mass ratio by UV irradiation. Exp. Cell Res. **95**, 405-415. (10.1016/0014-4827(75)90566-2) 1238278

[42] Tyson J, Garcia-Herdugo G, Sachsenmaier W. 1979 Control of nuclear division in *Physarum polycephalum*: comparison of cycloheximide pulse treatment, UV irradiation, and heat shock. Exp. Cell Res. **119**, 87-98. (10.1016/0014-4827(79)90338-0) 761604

[43] Zatulovskiy E, Skotheim JM. 2020 On the molecular mechanisms regulating animal cell size homeostasis. Trends Genet. **36**, 360-372. (10.1016/j.tig.2020.01.011) 32294416 PMC7162994

[44] Keifenheim D, Sun XM, D'Souza E, Ohira MJ, Magner M, Mayhew MB, Marguerat S, Rhind N. 2017 Size-dependent expression of the mitotic activator Cdc25 suggests a mechanism of size control in fission yeast. Curr. Biol. **27**, 1491-1497.E4. (10.1016/j.cub.2017.04.016) 28479325 PMC5479637

[45] Gerard C, Tyson JJ, Coudreuse D, Novak B. 2015 Cell cycle control by a minimal Cdk network. PLoS Comput. Biol. **11**, e1004056. (10.1371/journal.pcbi.1004056) 25658582 PMC4319789

[46] Goldbeter A. 2017 Dissipative structures and biological rhythms. Chaos **27**, 104612. (10.1063/1.4990783) 29092409

